# Prevalence and associated factors of insomnia among adults in Anhui Province, China

**DOI:** 10.3389/fpubh.2026.1794419

**Published:** 2026-05-08

**Authors:** Junwei Yan, Yong Liu, Xiaotong Xu, Hongyu Wang

**Affiliations:** 1 Science and Education Department, Affiliated Psychological Hospital of Anhui Medical University, Hefei, Anhui, China; 2 Psychiatry Department, Hefei Fourth People's Hospital, Hefei, Anhui, China; 3 Prevention and Control Department, Anhui Mental Health Center, Hefei, Anhui, China

**Keywords:** chronic diseases, female, insomnia, lower income, self-rated health

## Abstract

**Background:**

Insomnia is a highly prevalent sleep disorder with substantial public health implications. However, comprehensive data on its prevalence and associated factors in Anhui Province, China, are limited. This study aimed to investigate the prevalence of insomnia and its key influencing factors among adults in Anhui Province.

**Methods:**

A population-based cross-sectional study was conducted in 2021 across 15 cities in Anhui Province, involving 8,317 adults aged ≥18 years selected via multi-stage probability sampling. Insomnia symptoms were assessed using the Insomnia Severity Index (ISI), with a score of ≥7 indicating the presence of insomnia. Data on socio-demographics, lifestyle, and health status were collected via face-to-face interviews. Univariate and multivariate logistic regression analyses were employed to identify factors associated with insomnia.

**Results:**

The overall prevalence of insomnia was 13.95% (1,160/8317). Multivariate analysis revealed that female gender (*OR* = 1.415, 95% *CI*: 1.343–1.849), presence of chronic diseases (*OR* = 1.710, 95% *CI*: 1.470–1.988), and poorer self-rated health status (e.g., “poor” *vs.* “good”: *OR* = 6.593, 95% *CI*: 5.299–8.202) were significantly associated with higher odds of insomnia. A non-linear relationship was observed with personal monthly income, where the odds were lowest for the middle-income bracket (3000–5,000 CNY, *OR* = 0.629, 95% *CI*: 0.488–0.811, ref.: <1,500 CNY) and slightly increased but not statistically significant for the highest income group (≥5,000 CNY).

**Conclusion:**

Insomnia symptoms are common among adults in Anhui Province, affecting approximately one in seven individuals. The condition is strongly associated with being female, having chronic diseases, lower income, and poor self-rated health. These findings underscore the need to integrate sleep health screening and management into primary care and public health strategies, with particular attention to vulnerable subgroups.

## 1 Introduction

Insomnia is a highly prevalent sleep disorder with substantial public health implications worldwide. A recent systematic review estimated the global adult prevalence of clinically relevant insomnia to be 16.2%, affecting over 850 million people, with nearly half experiencing severe insomnia (1). This burden is comparable to other major global health conditions, reinforcing the need for comprehensive sleep health initiatives. Epidemiological estimates vary widely depending on case definitions, assessment tools, and sampling methods. A recent global meta-analysis confirmed insomnia as a common disorder, reporting a pooled prevalence of 12.4% when diagnosed via clinician-administered DSM interviews, and 16.3% when based on self-reported DSM criteria (2). The choice of diagnostic instrument significantly impacts estimates; for example, studies using the Athens Insomnia Scale (AIS) with a cut-off of 6 yielded a pooled prevalence of 32.3%, whereas using the Insomnia Severity Index (ISI) with a cut-off of 10 yielded 12.5%, closely aligning with interview-based rates (2). A large population-based study from Norway, applying different diagnostic manuals to the same cohort, reported insomnia prevalence ranging from 8.5% (DSM-5) to 23.6% (DSM-IV-TR), highlighting how stricter criteria requiring both sleep dissatisfaction and daytime impairment yield lower rates (3). This aligns with findings from a multi-country European study using the 2020 National Health and Wellness Survey (NHWS), which estimated the prevalence of chronic insomnia disorder (CID)-defined via proxy DSM-5-TR criteria-to be 6.0% across five European nations, with national rates ranging from 5.5 to 6.7% (4). A study from the UK Biobank similarly indicated a gap between symptom burden (29% with frequent insomnia symptoms) and clinical diagnosis in primary care (6%), underscoring issues of recognition and differing definitions (5). Insomnia is consistently more common among women, older adults (up to a certain age), and individuals with comorbid medical or psychiatric conditions (1, 2, 5, 6). Its course is often chronic, and insomnia is both a consequence and a precursor of various adverse outcomes, including depression, anxiety, cardiovascular diseases, metabolic disorders, and impaired daytime functioning (5, 7, 8). The humanistic and economic burden is substantial, with insomnia associated with poorer health-related quality of life, reduced work productivity, and increased healthcare resource utilization (4, 9).

Beyond its high prevalence, insomnia is increasingly recognized as a significant risk factor for major health conditions. A meta-analysis confirms that insomnia with objective short sleep duration (ISSD)-a phenotype reflecting greater physiological hyperarousal is associated with a higher risk of prevalent and incident hypertension compared to normal sleepers or those with insomnia but normal objective sleep duration (10). This association underscores the potential role of insomnia severity, indexed by objective sleep measures, in cardiovascular health. Furthermore, sleep disturbances are robustly linked to cognitive decline. A large multicenter study in China found that insomnia symptoms, particularly difficulties initiating sleep and daytime sleepiness, were associated with an increased risk of mild cognitive impairment (MCI) and poorer cognitive performance, with effects more pronounced in rural residents (11). This aligns with accumulating evidence suggesting that sleep is essential for brain function and clearance of neurodegenerative proteins. Critically, longitudinal research using trajectory modelling has demonstrated that sustained high levels of insomnia symptoms accelerate global cognitive decline, whereas recovery from insomnia (a decreasing symptom trajectory) is associated with a deceleration in cognitive decline (12). This highlights the dynamic and potentially modifiable nature of the insomnia-cognition relationship.

The aetiology of insomnia is multifactorial, involving biological, psychological, and social determinants. Socioeconomic factors, health behaviors, comorbidities, and self-rated health are consistently linked to insomnia risk (5, 6, 9). Female gender, lower income, and the presence of chronic physical or mental health conditions have been identified as strong correlates (1, 2, 5, 6). For instance, among occupational groups such as truck drivers, insomnia is significantly associated with a 2-fold increased risk of lifestyle-related diseases including hypertension and dyslipidemia (7). Shared mechanisms including genetic vulnerability, dysregulation of the hypothalamic–pituitary–adrenal axis, and hyperarousal are thought to underlie the comorbidity between insomnia and psychological/physical conditions (8, 10, 13). Cognitive-behavioral models further emphasize the role of conditioned wakefulness and maladaptive sleep-related behaviors in perpetuating the disorder (8).

Recent studies across diverse populations and contexts provide additional insights. Longitudinal data from Japan, for example, show that insomnia-related symptoms (IRS) among adults were lowest in 1995, peaked in 2007, and then declined through 2013, with notable socioeconomic disparities linked to employment status and marital status (14). This trend underscores how macroeconomic shifts and social structures can influence population sleep health over time. In other settings, among postpartum mothers in Mattu City, Ethiopia, the prevalence of insomnia was 29.3%, with risk factors including primiparity, unplanned pregnancy, lack of postnatal follow-up, intimate partner violence, partner alcohol use, and partner dissatisfaction with the infant’s gender (15). A large-scale study of adults in Guangdong, China, reported a weighted insomnia prevalence of 24.8%. This study highlighted strong associations between insomnia and depression and anxiety, and identified female gender, higher education (10–16 years), chronic diseases, and alcohol use as risk factors, while being married/cohabiting and older age were protective (16). The European NHWS analysis also found high comorbidity of CID with pain, anxiety, and depression, and that insomnia severity was a strong predictor of worse outcomes (4).

Specific patient groups with neurological disorders are at particularly high risk. A meta-analysis found that 44% of patients with Parkinson’s disease (PD) experience insomnia, and its presence is associated with longer disease duration, higher medication load, and more severe depressive symptoms (17). In populations exposed to extreme stress, such as people with epilepsy (PWE) in Jordan during the Gaza war, the prevalence of severe insomnia reached 51.8%, and was associated with female gender and not abstaining from social media exposure to war-related content (18). Specific occupational groups also show elevated risk; among firefighters in the Dhaka division of Bangladesh, 22.9% reported moderate to severe insomnia (19). Among geriatric populations in Bangladesh, the prevalence of insomnia was found to be exceptionally high at 77% (20). This underscores the profound impact of psychological trauma and media exposure on sleep.

In the context of the COVID-19 pandemic, studies indicate a surge in sleep disturbances. A meta-analysis of 22 studies found that the pooled prevalence of insomnia symptoms among patients with COVID-19 was 48%, with depression and anxiety symptoms also highly prevalent at 38% each (21). Research on long COVID patients revealed that clinically significant insomnia was present in 50% of patients approximately 6 months post-infection and 42% one year post-infection, strongly correlated with symptoms of anxiety, depression, and PTSD rather than the initial severity of the infection (22). A national survey of mental health professionals in China who recovered from COVID-19 found an insomnia prevalence of 36.2%, with symptoms intricately networked with depression and suicidality (23). Similarly, among older Chinese adults during the COVID-19 outbreak, the one-month prevalence of any insomnia symptom was 23.4% (24). In other settings, such as Mettu town, Ethiopia, during COVID-19 lockdowns, insomnia symptom prevalence reached 52.6%, associated with female gender, smoking, fear of COVID-19, and psychological distress (25).

Given the lack of comprehensive local data, the impact of diagnostic criteria variation, and the expanding understanding of socioeconomic factors, this study aimed to investigate insomnia prevalence and its key influencing factors among residents in 15 cities of Anhui Province, providing a scientific basis for targeted prevention and intervention strategies aligned with global guidelines.

## 2 Methods and materials

### 2.1 Research design and subjects

From October to November 2021, a cross-sectional study was conducted in Anhui Province, located in the Yangtze River Delta region of eastern China. Anhui Province comprises 16 prefecture-level cities, stretching north to south. The province is divided into three regions-Northern Anhui, Central Anhui, and Southern Anhui-by the Huai River and Yangtze River. The northern region comprises plains, encompassing Fuyang, Bozhou, Bengbu, Suzhou, Huaibei, and Huainan; the central region consists of hilly terrain, including Anqing, Hefei, Chuzhou, and Lu′an; while the southern region features mountainous areas, covering Chizhou, Tongling, Wuhu, Ma’anshan, Xuancheng, and Huangshan.

Permanent urban and rural residents aged ≥18 years who had resided locally for over 6 months were selected as study subjects after signing informed consent forms. The exclusion criteria were as follows: (1) individuals who refused to provide written informed consent after repeated explanation; (2) individuals who could not be contacted after three separate home visits; (3) individuals with severe cognitive impairment or mental illness that precluded understanding or completion of the interview; (4) individuals who were bedridden or unable to communicate verbally; and (5) temporary residents (e.g., visitors) who did not meet the residency requirement.

Sample size calculation followed the formula $n=\frac{z_{\alpha /2}^{2}\times (1-p)}{\varepsilon^{2}p}$ for sampling surveys, with α = 0.05, ε = 0.06, and referencing China’s 2016 insomnia prevalence rate *p* = 15%. Considering an estimated 25% non-response rate (including invalid questionnaires and refusal to participate), the minimum sample size was determined to be 8,063 individuals.

### 2.2 Sampling methodology

Stage 1—Selection of counties (districts) and sub-districts (towns): Using probability proportional to size (PPS) sampling, 30 counties (districts) were randomly selected from across Anhui Province. For each selected county (district), two sub-districts (towns) were then randomly chosen using a random number table, yielding a total of 60 sub-districts (towns) province-wide.

Stage 2—Selection of neighborhood (village) committees: Within each selected sub-district (town), the names of all neighborhood (village) committees and the total number of households were collected. Using a random number method, three neighborhood (village) committees were selected from each sub-district (town), resulting in 180 committees province-wide.

Stage 3—Selection of households: Vacant dwellings, commercial-residential properties, and invalid addresses were first excluded from each selected neighborhood (village) committee. Systematic sampling was then applied to determine the starting point and sampling interval, and 47 households were selected from each committee.

**Figure 1:**
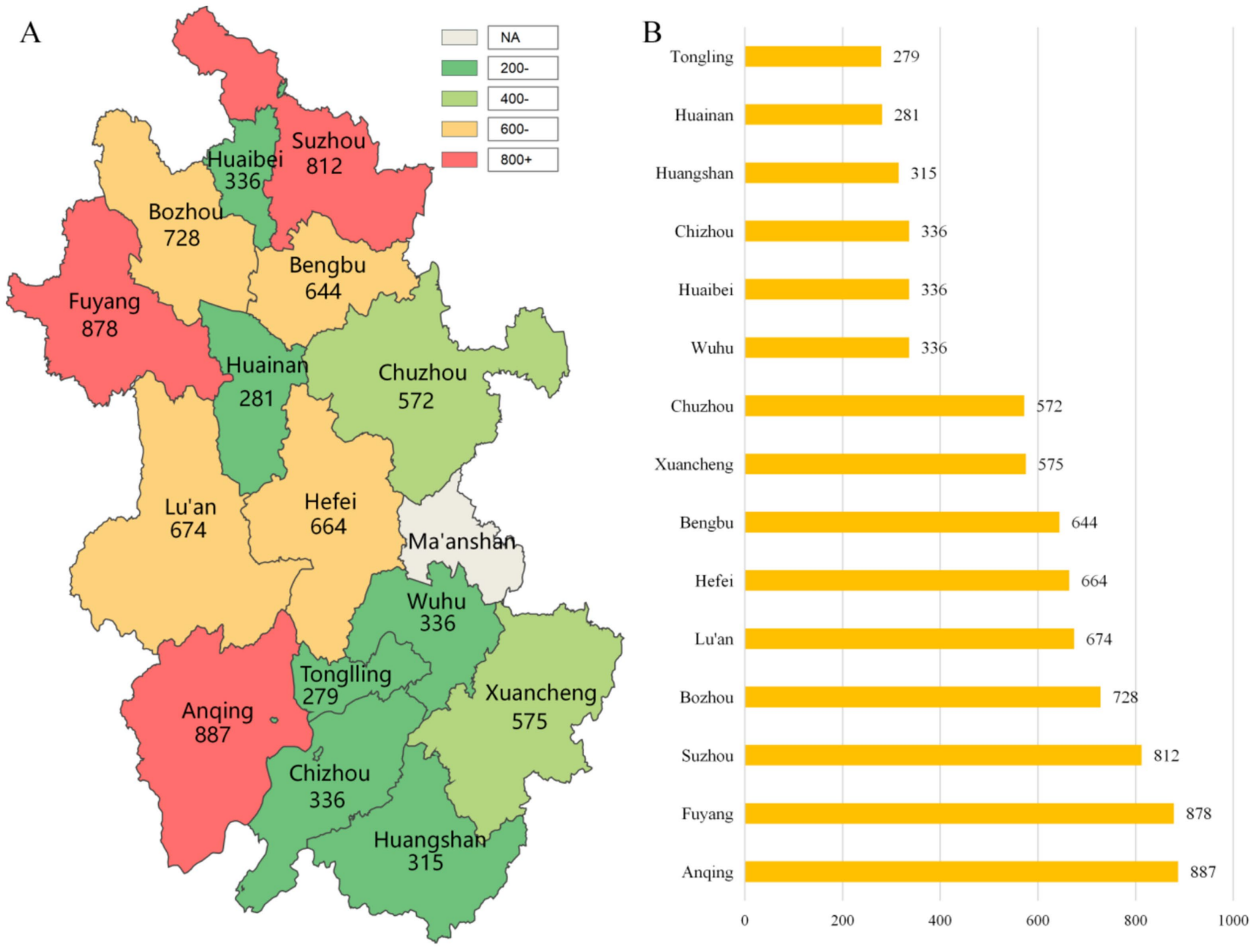
Distribution of Sampling Results Across 15 Cities in Anhui Province, China. **(A)** Color-coded map showing the number of sampled individuals per city (values ranging from 279 to 887, with city names labeled). **(B)** Horizontal bar chart presenting the sample sizes in ascending order.

Stage 4—Selection of survey respondents: All residents aged ≥18 years in the selected households were registered, and their age and gender were recorded. At the provincial level, one individual per household was selected as the survey respondent using simple random sampling. Of the 8,460 people sampled across the province, 143 individuals (1.69%) did not provide valid responses. Among them, 98 refused to participate, 32 could not be reached after three home visits, and 13 did not complete the questionnaire. Therefore, a total of 8,317 valid questionnaires were completed from 15 cities and were included in the comprehensive case analysis (26), yielding a response rate of 98.31%. The detailed distribution is shown in Figure 1.

### 2.3 Measures

This survey was conducted using paper questionnaires by investigators trained in consistency. Face-to-face interviews were administered through centralized or door-to-door visits. Each interview lasted approximately 20–30 min. Interviewers followed a standardized script to address participant questions uniformly, ensuring consistency across all sessions. The questionnaire covered demographic characteristics (home address, gender, age, education level, marital status, and total monthly household income), lifestyle factors (alcohol consumption and smoking status), and self-reported health status. The Insomnia Severity Index (ISI) is a reliable and effective tool for quantifying the subjective severity of insomnia (27). This 7-item scale assesses the severity of insomnia and its impact over the past month, with the Chinese version having undergone reliability and validity testing in Chinese population (28–30). Each item is scored on a 0–4 Likert scale, with the total score calculated by summing all item scores. The total score ranges from 0 to 28 points. Following the approach validated by Chung et al. to maximize screening sensitivity in community-based epidemiological studies, a score of 7 or higher was used to define the presence of insomnia symptoms (28). We assessed the internal consistency of ISI in our sample, which yielded a Cronbach’s *α* coefficient of 0.924, indicating excellent internal consistency.

### 2.4 Quality control

To ensure data quality throughout the survey, we implemented multi-stage quality control procedures. Pre-survey: provincial staff trained all field investigators using a standardized protocol; sampling was completed by the provincial team and remained unchanged thereafter. During the survey: fieldworkers followed a standardized protocol; substitution with demographically similar individuals was permitted only when the original participant was unreachable or persistently refused, with the substitution rate capped at 8% and recorded. County-level supervisors accompanied fieldworkers during the initial days to monitor performance, and municipal supervisors conducted at least two on-site visits. Weekly progress reports were submitted for timely supervision. Post-survey: original paper questionnaires and electronic databases were reviewed at county, municipal, and provincial levels. Quality control staff randomly selected 10, 5, and 1% of completed questionnaires at each level, respectively, for verification through on-site or telephone review, checking for completeness, logical consistency, and adherence to the sampling protocol. If more than five invalid questionnaires were identified at a given site, the site was required to re-conduct the survey.

### 2.5 Statistical analysis

The conversion of residential address coordinates and statistical map generation were both accomplished using DataMap for Excel (Version 9.2.2.0). Data processing utilized SPSS 22.0 software. Categorical variables were described using frequencies and percentages. Continuous variables (e.g., age) were summarized as mean ± standard deviation. Binary logistic regression analysis was conducted to examine differences in insomnia across different demographic groups. Univariate logistic regression was first performed for each potential factor; variables with *p* < 0.05 in univariate analysis were entered into multivariate logistic regression using the forward Wald method, with entry criterion set at *p* < 0.05/N, (where N is the number of variables included in the univariate analysis, i.e., *Bonferroni* correction) and removal criterion set at *p* > 0.05. This conservative threshold was applied to control for multiple comparisons (31). All variables listed in Table 1 were considered potential confounders and were entered into the multivariate logistic regression model. To assess robustness, we also conducted a forward stepwise (likelihood ratio) multivariate model including all candidate variables; the results were essentially unchanged (data not shown). Odds ratios (OR) and 95% confidence intervals (CI) were reported. To evaluate the overall model fit of the multivariate logistic regression, we reported the Nagelkerke *R^2^*, which indicates the proportion of variance in the insomnia outcome explained by the included predictors. To assess the robustness of our findings with respect to the choice of ISI threshold, we conducted a sensitivity analysis by redefining insomnia using higher thresholds (ISI ≥ 8, ≥10 and ≥15). For each threshold, we calculated the prevalence of insomnia and reran the multivariate logistic regression model incorporating the same set of potential risk factors.

**Table 1:** General demographic data.

Variable	Number	Percentage (%)
Regional distribution		
Northern Anhui	3,679	44.23
Central Anhui	2,797	33.63
Southern Anhui	1841	22.14
Gender		
Male	3,955	47.55
Female	4,362	52.45
Age		
18 ~ <40	2,650	31.86
40 ~ <60	3,315	39.86
≥60	2,352	28.28
Education level		
Junior High School or Below	6,429	77.30
High School/Vocational High School/Technical Secondary School	1,011	12.16
College/Undergraduate or Higher	876	10.53
Occupation		
Civil Servant/Professional Technician	766	9.21
Business/Service Industry	1,172	14.09
Farmer/Worker	6,016	72.33
Other	360	4.33
Marital status		
Unmarried	860	10.34
Married	6,925	83.26
Divorced/Widowed/Other	532	6.40
Chronic illness		
No	4,845	58.25
Yes	3,472	41.75
Personal monthly income (CNY)		
<1,500	4,227	50.82
1,500 ~ <3,000	1903	22.88
3,000 ~ <5,000	1,533	18.43
≥5,000	654	7.86
Monthly household income (CNY)		
<3,000	2,575	30.96
3,000 ~ <6,000	2,539	30.53
6,000 ~ <10,000	1937	23.29
≥10,000	1,266	15.22
Smoker		
No	6,441	77.44
Yes	1876	22.56
Alcohol consumer		
No	6,062	72.89
Yes	2,255	27.11
Self-rated health status		
Good	5,474	65.82
Average	2,307	27.74
Poor	536	6.44

## 3 Results

### 3.1 General demographic characteristics

Among the 8,317 respondents, regional distribution showed 3,679 (44.23%) individuals from northern Anhui, 2,797 (33.63%) individuals from central Anhui, and 1841 (22.14%) individuals from southern Anhui. Gender distribution indicated 3,955 (47.55%) males and 4,362 (52.45%) females. The average age was 48.91 ± 15.89 years. Educational attainment was junior high school or below for 6,429 (77.30%) individuals. Occupation was farmer/worker for 6,016 (72.33%) individuals. Marital status was married for 6,925 (83.26%) individuals. 3,472 (41.75%) individuals had chronic diseases. 4,227 (50.82%) individuals had a monthly personal income below ¥1,500, and over 60% of respondents had monthly household incomes below ¥6,000. 1876 (22.56%) individuals were smoker, and 2,255 (27.11%) individuals were alcohol consumer. 5,474 (65.82%) individuals self-reported good health status. Other basic demographic information is detailed in Table 1.

### 3.2 Insomnia prevalence

**Figure 2:**
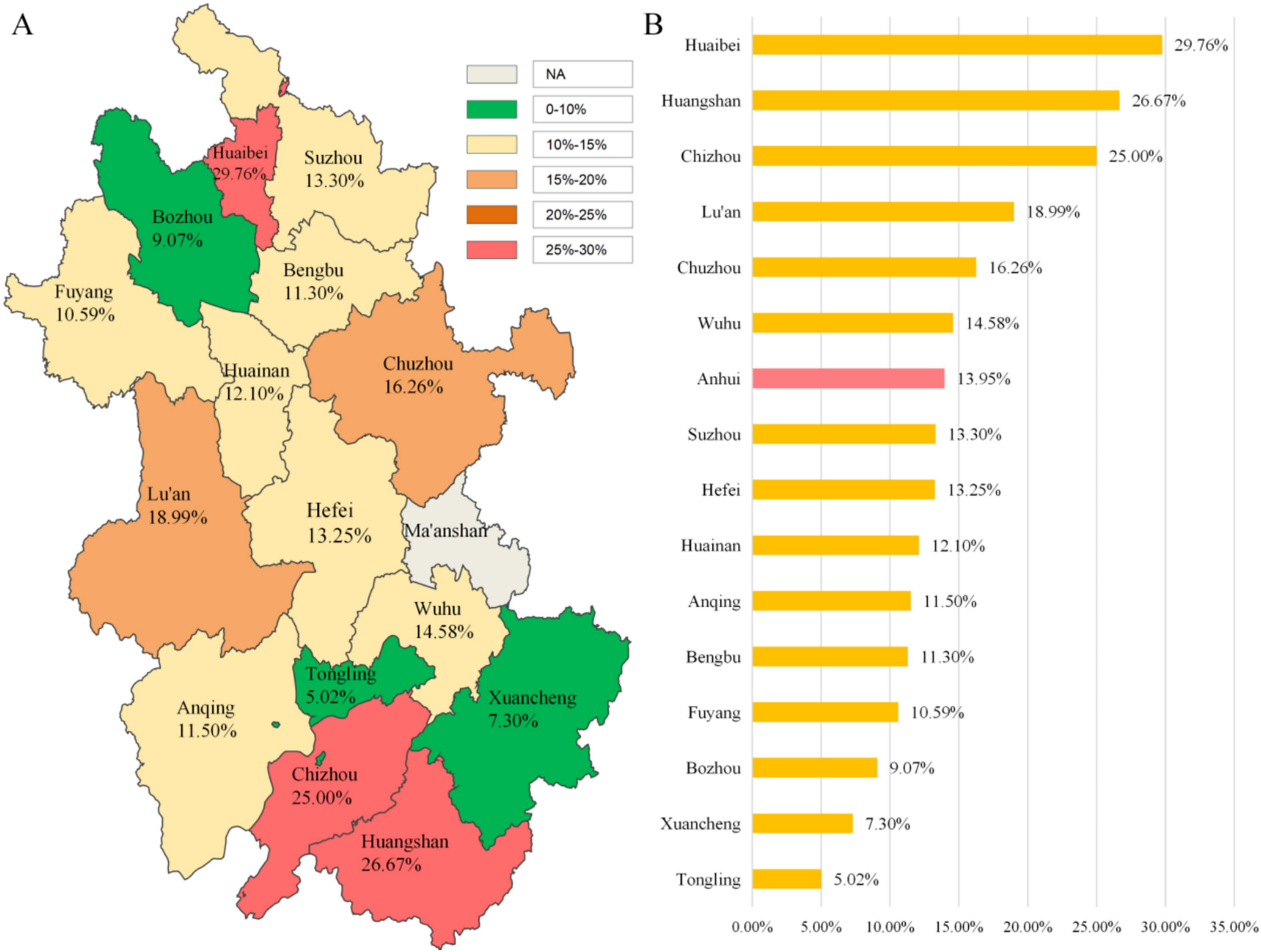
Prevalence of Insomnia among Adults in Anhui Province, China. **(A)** Color-coded map displayingthe insomniaprevalence (%) per city. **(B)** Horizontal bar chart presenting the prevalence rates in descending order.

Among the 8,317 survey participants, 1,160 reported insomnia, resulting in an insomnia prevalence of 13.95% in Anhui Province. Among the 15 cities included in the survey, six cities had insomnia prevalence rates higher than the provincial average, while nine cities had rates lower than the provincial average. Specific prevalence rates are detailed in Figure 2.

Following sensitivity analyses using higher ISI thresholds, the estimated prevalence gradually decreased: 10.64% (95% CI: 10.00–11.30%) when ISI ≧ 8, 7.33% (95% CI: 6.78–7.89%) when ISI ≧ 10, and 2.57% (95% CI: 2.23–2.92%) when ISI ≧ 15. However, as shown in Supplementary Table S1, the direction and significance of the association between all identified risk factors (gender, chronic diseases, income, self-rated health, etc.) and insomnia remained consistent across all thresholds. When the thresholds were set at 8, 10 and 15, the Nagelkerke *R^2^* values were 0.173, 0.173 and 0.180 respectively, indicating that the model’s explanatory power remained stable. The results are presented in Supplementary Tables S1, S2.

### 3.3 Analysis of factors affecting insomnia

Univariate analysis revealed statistically significant differences in insomnia rates across various demographic factors including gender, age, educational attainment, occupation, marital status, presence of chronic diseases, personal monthly income, household monthly income, smoking status, alcohol consumption, and self-rated health status (*p* < 0.05). However, no statistically significant differences were observed in insomnia prevalence between the southern, northern, and central regions of Anhui Province (*p* > 0.05). For more detailed results, see Table 2.

**Table 2:** Univariate analysis of factors influencing insomnia prevalence among adults in Anhui Province.

Variable	Number	Percentage (%)	Number of insomniacs	Insomnia rate (%)	*OR*(95%*CI*)	*P*
Regional distribution						0.061
Northern Anhui	3,679	44.23	476	12.94	1	
Central Anhui	2,797	33.63	411	14.69	1.159(1.005, 1.336)	0.042
Southern Anhui	1841	22.14	273	14.83	1.172(0.998, 1.376)	0.053
Gender						<0.001
Male	3,955	47.55	461	11.66	1	
Female	4,362	52.45	699	16.02	1.446(1.275–1.641)	<0.001
Age						
18–<40	2,650	31.86	207	7.81	1	
40–<60	3,315	39.86	482	14.54	2.008(1.691–2.384)	<0.001
≥60	2,352	28.28	471	20.30	2.955(2.483–3.517)	<0.001
Education level						<0.001
Junior High School or Below	6,429	77.30	982	15.27	1	
High School/Vocational High School/Technical Secondary School	1,011	12.16	86	8.51	0.516(0.409–0.650)	<0.001
College/Undergraduate or Above	876	10.53	92	10.50	0.651(0.519–0.816)	<0.001
Occupation						<0.001
Civil Servant/Professional Technician	766	9.21	62	8.09	1	
Business/Service Industry	1,172	14.09	149	12.71	1.654(1.211–2.258)	0.002
Farmers/Workers	6,016	72.33	928	15.43	2.071(1.583–2.710)	<0.001
Other	360	4.33	21	5.83	0.703(0.422–1.173)	0.178
Marital status						<0.001
Unmarried	860	10.34	72	8.37	1	
Married	6,925	83.26	965	13.94	1.772(1.379–2.277)	<0.001
Divorced/Widowed/Other	532	6.40	123	23.12	3.291(2.403–4.507)	<0.001
Chronic illness						<0.001
No	4,845	58.25	408	8.42	1	
Yes	3,472	41.75	752	21.66	3.007(2.641–3.423)	<0.001
Personal monthly income (CNY)						<0.001
<1,500	4,227	50.82	776	18.36	1	
1,500 ~ <3,000	1903	22.88	196	10.30	0.511(0.432–0.603)	<0.001
3,000 ~ <5,000	1,533	18.43	114	7.44	0.357(0.291–0.439)	<0.001
≥5,000	654	7.86	74	11.31	0.567(0.440–0.732)	<0.001
Monthly household income (CNY)						<0.001
<3,000	2,575	30.96	499	19.38	1	
3,000 ~ <6,000	2,539	30.53	287	11.30	0.530(0.453–0.620)	<0.001
6,000 ~ <10,000	1937	23.29	203	10.48	0.487(0.409–0.580)	<0.001
≥10,000	1,266	15.22	171	13.51	0.650(0.538–0.784)	<0.001
Smoker						0.003
No	6,441	77.44	937	14.55	1	
Yes	1876	22.56	223	11.89	0.792(0.678–0.926)	0.003
Alcohol consumer						0.859
No	6,062	72.89	843	13.91	1	
Yes	2,255	27.11	317	14.06	1.013(0.881–1.164)	0.859
Self-rated health status						<0.001
Good	5,474	65.82	403	7.36	1	
Fair	2,307	27.74	525	22.76	3.707(3.221–4.267)	<0.001
Poor	536	6.44	232	43.28	9.603(7.872–11.714)	<0.001

Factors with statistically significant results from the univariate analysis were incorporated into a multivariate logistic regression equation. Ultimately, four factors-gender, chronic illness, personal monthly income and self-rated health status-were included in the equation. The Nagelkerke *R^2^* was 0.156, indicating that the model explained 15.6% of the variance in insomnia status. Among these, the insomnia rate among women was higher than that among men (16.02% *vs*. 11.66%, *OR* = 1.415, 95%*CI*: 1.343–1.849), and the insomnia rate among individuals with chronic diseases was higher than among those without chronic diseases (21.66% *vs.* 8.42%, *OR* = 1.710, 95%*CI*: 1.470–1.988). Compared to individuals earning less than 1,500 yuan monthly, those with monthly incomes between 3,000 and 5,000 yuan exhibit a significantly lower incidence of insomnia (7.44% *vs*. 18.36%, *OR* = 0.629, 95%*CI*: 0.488–0.811). However, no significant difference was observed between the highest income group (≧5,000 yuan) and the reference group (11.31% *vs.* 18.36%, *OR* = 0.907, 95%*CI*: 0.659–1.249). The prevalence of insomnia increases progressively from good to poor self-rated health status. Compared with those who rated their health as good, individuals who rated their health as poor and fair showed a significantly higher prevalence of insomnia (43.28% *vs*. 7.36%, *OR* = 6.593, 95%*CI*: 5.299–8.202; 22.76% *vs*. 7.36%, *OR* = 2.963, 95%*CI*: 2.547–3.447). The results were presented in Table 3.

**Table 3:** Multifactorial analysis of factors influencing insomnia prevalence among adults in Anhui Province.

Variable	Number	Percentage (%)	Number of insomniacs	Insomnia rate (%)	*OR*(95%*CI*)	*P*
Gender						<0.001
Male	3,955	47.55	461	11.66	1	
Female	4,362	52.45	699	16.02	1.415(1.232–1.624)	<0.001
Chronic illness						<0.001
No	4,845	58.25	408	8.42	1	
Yes	3,472	41.75	752	21.66	1.710(1.470–1.988)	<0.001
Personal monthly income (CNY)						<0.001
<1,500	4,227	50.82	776	18.36	1	
1,500 ~ <3,000	1903	22.88	196	10.30	0.814(0.668–0.991)	0.022
3,000 ~ <5,000	1,533	18.43	114	7.44	0.629(0.488–0.811)	<0.001
≥5,000	654	7.86	74	11.31	0.907(0.659–1.249)	0.551
Self-rated health status						<0.001
Good	5,474	65.82	403	7.36	1	
Fair	2,307	27.74	525	22.76	2.963(2.547–3.447)	<0.001
Poor	536	6.44	232	43.28	6.593(5.299–8.202)	<0.001

## 4 Discussion

This population-based study in Anhui Province, China, found an overall insomnia prevalence of 13.95% among adults. This rate is lower than the recent global estimate of 16.2% for clinically relevant insomnia (1). It is higher than the 6% prevalence of chronic insomnia disorder (CID) reported across five European countries using proxy DSM-5-TR criteria in the 2020 NHWS (4) and the 6% prevalence of clinically recorded insomnia in a large English cohort (5). It aligns closely with the pooled prevalence of 12.4% for DSM-based diagnostic interviews reported in a recent global meta-analysis (2). However, it is lower than the 16.3% pooled prevalence for studies using self-reported DSM criteria from the same meta-analysis, which may be a more comparable method to the simple self-report item used in our study (2). Our rate is also lower than rates reported in other Chinese studies, such as the 24.8% found in Guangdong Province (16) and the 23.4% among older adults during COVID-19 (24). The rate is substantially lower than that reported among specific clinical and high-stress populations, including patients with Parkinson’s disease (44%) (17), people with epilepsy during war (51.8%) (18), patients with COVID-19 (48%) (21), postpartum mothers in Ethiopia (29.3%) (15), firefighters in Bangladesh (22.9%) (19), geriatric populations in Bangladesh (77%) (20), and Chinese mental health professionals post-COVID-19 (36.2%) (23).

Such discrepancies are common in insomnia epidemiology and can be attributed to several factors underscored by recent research. First, the assessment method is paramount. Our study used the ISI which is a validated tool used in the European burden analysis (4), but not the strict diagnostic criteria (e.g., DSM-IV, ICSD-2/3) applied in the global systematic review (1). The recent meta-analysis clearly demonstrates that prevalence estimates vary dramatically depending on the instrument and cut-off used, with differences exceeding 20 percentage points between methods (2). Studies employing stricter diagnostic criteria (e.g., DSM-5 requiring both night-time symptoms and daytime impairment via clinical interview or detailed proxy questions) yield lower prevalence estimates. Second, differences in study populations greatly influence rates. Populations with chronic health conditions or exposure to major stressors consistently report higher rates (7, 17, 18, 21). Finally, regional socioeconomic, cultural, and contextual factors also play a role, as evidenced by significant geographical variations in prevalence within the global analysis (1, 2, 5, 6, 16). The observed trends in Japan, where IRS prevalence changed over time in relation to economic conditions (14), further highlight the importance of temporal and macroeconomic contexts.

The identified risk factors in our study align robustly with international and regional evidence. The higher prevalence among women is a consistent finding across diverse populations and study designs, from the global review (1) to Bangladesh (20) and Ethiopia (15, 25) to multinational studies (2, 5, 6), other Chinese cohorts (16), Japanese adults (14), and the European CID population (4). The strong association with chronic diseases underscores the well-established bidirectional relationship between poor sleep and physical health, a pattern widely confirmed in other settings, including among patients with Parkinson’s disease (17). This link extends to specific morbidities, for instance, insomnia with objective short sleep duration is identified as a phenotype associated with a significantly higher risk of hypertension (10). Notably, our study did not assess mental health comorbidities like depression or anxiety, which were highly prevalent and showed extremely strong associations with insomnia in the Guangdong study (16). This represents a significant limitation, as these unmeasured confounders may influence or partly explain the observed associations with factors like self-rated health and chronic disease.

The observation that poorer self-rated health was the strongest correlate of insomnia emphasizes the profound impact of sleep disturbances on perceived well-being and daytime function. This aligns with clinical insights that insomnia significantly impairs quality of life (8) and is consistent with findings from other settings where poor self-rated health was a key predictor (24, 32), and with the documented humanistic burden of CID on HRQoL measures like EQ-5D-5L (4). The association between personal income and insomnia did not follow a strict gradient pattern. The decrease in prevalence from the lowest (<1,500 CNY) to the mid-level income bracket (3000–5,000 CNY) may reflect reduced financial stress. The slight, non-significant uptick at the highest income level (≥5,000 CNY) could be related to job-related stressors associated with higher-income occupations-a nuance observed in other contexts where high occupational demands disrupt sleep (5, 33). This complex gradient suggests that both material deprivation and high-pressure prosperity can be risk factors for sleep disruption, a finding that warrants further exploration in different occupational and cultural settings. It is interesting to contrast this with the Japanese study, where household expenditure was not consistently associated with IRS, possibly due to Japan’s universal health system and lower health inequalities (14).

In addition to their individual effects, these factors may also influence the risk of insomnia through complex interactions. For example, the close association between chronic illness and insomnia likely reflects a bidirectional relationship: chronic pain or illness can disrupt sleep architecture, whilst poor sleep quality can in turn exacerbate disease symptoms through inflammation, neuroendocrine and autonomic dysregulation (10). Furthermore, individuals with chronic conditions often experience psychological distress, which may partially mediate the impact of chronic illness on insomnia. A lower self-rated health score (the strongest predictor in this study) may not only reflect physical health status but also reveal underlying symptoms of depression or anxiety-factors known to be highly comorbid with insomnia (16). Women report poorer self-rated health and have a higher prevalence of chronic pain; furthermore, due to differences in coping strategies and help-seeking behaviors, they may be more susceptible to stress-related sleep disorders (2). Consequently, the observed associations are likely the result of intertwined biological, psychological and social mechanisms, rather than isolated causal pathways. Future longitudinal studies incorporating mediation analyses are required to elucidate these complex interactions.

There is one further observation that warrants brief discussion. In univariate analysis, the prevalence of insomnia appeared to be lower among smokers. This finding appears counterintuitive, as the majority of the literature indicates that smoking is a risk factor for insomnia (34). The univariate results in this study are likely explained by confounding factors related to demographic characteristics. In the study sample, smokers were predominantly male (94.03% of smokers were male), whereas in this population, male sex was associated with a lower prevalence of insomnia. Indeed, after adjusting for sex in the multivariate analysis, smoking was no longer significantly associated with insomnia and was not included in the final model. This pattern is consistent with previous studies, which have identified sex as important confounders in the relationship between smoking and insomnia (35). Furthermore, emerging evidence from Mendelian randomization studies suggests a bidirectional relationship between smoking and insomnia, with some evidence indicating that insomnia may lead to increased smoking, rather than the reverse (36). Nevertheless, the available evidence generally supports the view that smoking is a minor risk factor for insomnia, and the findings of this study should not be interpreted as suggesting that smoking has a protective effect.

Our findings should be interpreted in light of growing evidence on the long-term consequences of insomnia, particularly for cognitive health. Recent longitudinal research has moved beyond baseline assessments to model the trajectories of insomnia symptoms, revealing that sustained high insomnia symptoms accelerate global cognitive decline, while recovery from insomnia (a decreasing symptom trajectory) is associated with a slower rate of cognitive decline (12). This underscores the importance of not only identifying individuals with insomnia but also monitoring its course, as improving sleep may confer cognitive benefits. This aligns with findings from other studies linking insomnia symptoms to increased risk of cognitive impairment (11).

The findings have implications for management and public health. First-line treatment for chronic insomnia, as recommended by major guidelines, is Cognitive Behavioral Therapy for Insomnia (CBT-I), which has demonstrated durable long-term effects (8). Pharmacological options exist but are generally recommended for short-term use or when CBT-I is not available or effective. The strong link between insomnia, chronic disease, poor self-rated health, cognitive impairment, and the associated economic burden from work productivity loss and healthcare use highlights the need to integrate sleep health screening and management into primary care, chronic disease management, and geriatric care pathways. Furthermore, the association with lower income suggests that public health interventions should be accessible and affordable to reduce disparities. The global review reinforces the urgent need for comprehensive public health and clinical sleep health initiatives worldwide, given the high prevalence and significant burden identified (1).

Several limitations should be acknowledged. First, the cross-sectional design precludes causal inferences, a limitation shared with other large-scale surveys. Second, and most importantly, we did not assess mental health-related variables, particularly depression and anxiety. These conditions are closely associated with insomnia and may confound the associations we observed between chronic diseases, self-rated health status and insomnia. The extremely high odds ratio (OR = 6.59) for poor self-rated health may partly reflect unmeasured comorbidity with mental disorders, rather than being directly attributable solely to subjectively perceived physical health status. Therefore, the findings of this study should not be interpreted as evidence of an independent causal effect of the identified factors. Instead, they should be regarded as descriptive associations that may be influenced by underlying psychological distress. Third, potential interactions among risk factors (e.g., gender and chronic disease) were not examined due to the exploratory nature of the analysis and sample size considerations; future studies with larger samples could explore such interactions. Fourth, although we implemented rigorous investigator training and multi-stage quality control measures, we did not conduct formal inter-rater reliability testing (e.g., Kappa statistics) due to logistical constraints and limited resources for replicate assessments in this large-scale field survey. However, the high internal consistency of the ISI (Cronbach’s *α* = 0.924) and the comprehensive quality control procedures support the reliability of our data. Future studies should consider incorporating replicate assessments to directly quantify inter-rater agreement. Fifth, A sensitivity analysis using higher ISI thresholds (≧8, ≧10, and ≧15) confirmed that, although the estimated absolute prevalence varied as expected, the association between risk factors and insomnia remained robust and was not affected by the choice of threshold. This supports the validity of our main conclusions.

In conclusion, this study confirms a significant burden of insomnia symptoms in the general adult population of Anhui Province and identifies key sociodemographic and health-related correlates. The prevalence rate is lower than rates found in many other regional and high-risk populations. The findings reinforce that insomnia is a common condition intricately linked with female gender, economic factors, physical health, and self-perceived well-being, patterns that are consistent with the global profile. Future longitudinal studies incorporating objective sleep measurements to identify severe phenotypes, and detailed evaluation of mental, cognitive, and physical health comorbidities are needed to elucidate causal pathways and evaluate targeted interventions within the Chinese context.

## Data Availability

The raw data supporting the conclusions of this article will be made available by the authors, without undue reservation.

## References

[1] Benjafield AV Sert Kuniyoshi FH Malhotra A Martin JL Morin CM Maurer LF . Estimation of the global prevalence and burden of insomnia: a systematic literature review-based analysis. Sleep Med Rev. (2025) 82:102121. doi: 10.1016/j.smrv.2025.102121, 40627924 PMC12676268

[2] Van Straten A Weinreich KJ Fábián B Reesen J Grigori S Luik AI . The prevalence of insomnia disorder in the general population: a Meta-analysis. J Sleep Res. (2025) 34:e70089. doi: 10.1111/jsr.70089, 40369835 PMC12426706

[3] Porcheret K Hopstock LA Nilsen KB. Prevalence of insomnia in a general adult population cohort using different diagnostic criteria: the seventh survey of the Tromsø study 2015–2016. Sleep Med. (2024) 119:289–95. doi: 10.1016/j.sleep.2024.05.002, 38718598

[4] Chalet F-X Albanese E Egea Santaolalla C Ellis JG Ferini-Strambi L Heidbreder A . Epidemiology and burden of chronic insomnia disorder in Europe: An analysis of the 2020 National Health and wellness survey. J Med Econ. (2024) 27:1309–20. doi: 10.1080/13696998.2024.2407631, 39318277

[5] De Lange MA Richmond RC Eastwood SV Davies NM. Insomnia symptom prevalence in England: a comparison of cross-sectional self-reported data and primary care records in the UK biobank. BMJ Open. (2024) 14:e080479. doi: 10.1136/bmjopen-2023-080479, 38719300 PMC11086527

[6] Aernout E Benradia I Hazo J-B Sy A Askevis-Leherpeux F Sebbane D . International study of the prevalence and factors associated with insomnia in the general population. Sleep Med. (2021) 82:186–92. doi: 10.1016/j.sleep.2021.03.028, 33957414

[7] Ando T Miyachi T Sugano Y Kamatsuka M Mishima K Nomura K. The relationship between insomnia and lifestyle-related diseases among Japanese male truck drivers. Tohoku J Exp Med. (2023) 261:1–11. doi: 10.1620/tjem.2023.J052, 37344417

[8] Perlis ML Posner D Riemann D Bastien CH Teel J Thase M. Insomnia. Lancet. (2022) 400:1047–60. doi: 10.1016/S0140-6736(22)00879-0, 36115372

[9] Morin CM Jarrin DC. Epidemiology of insomnia. Sleep Med Clin. (2022) 17:173–91. doi: 10.1016/j.jsmc.2022.03.003 35659072

[10] Dai Y Vgontzas AN Chen L Zheng D Chen B Fernandez-Mendoza J . A meta-analysis of the association between insomnia with objective short sleep duration and risk of hypertension. Sleep Med Rev. (2024) 75:101914. doi: 10.1016/j.smrv.2024.101914, 38442466

[11] Liu X Zhou J Cheng G Yang M Hu F Liu D . Association between insomnia and its symptoms and cognitive impairment in community-dwelling older adults in China: a multicenter study. J Alzheimer's Dis. (2025) 106:1425–35. doi: 10.1177/13872877251353119, 40635465

[12] Huang Q-M Chen H Zhang P-D Yang J Liu D Li Z-H . Association between longitudinal trajectories of insomnia symptoms and subsequent cognitive decline: a prospective cohort study. Age Ageing. (2025) 54:afaf186. doi: 10.1093/ageing/afaf186, 40613668 PMC12231569

[13] Riemann D Krone LB Wulff K Nissen C. Sleep, insomnia, and depression. Neuropsychopharmacology. (2020) 45:74–89. doi: 10.1038/s41386-019-0411-y, 31071719 PMC6879516

[14] Otsuka Y Takeshima O Itani O Kaneko Y Suzuki M Matsumoto Y . Trends and socioeconomic inequities in insomnia-related symptoms among Japanese adults from 1995 to 2013. J Affect Disord. (2023) 323:540–6. doi: 10.1016/j.jad.2022.11.056, 36462611

[15] Alemu SS Wedajo LF Gezimu W Geda B Jarso MH. Prevalence of insomnia and associated factors among postpartum mothers in Mattu City, Southwest Ethiopia: a community-based study. BMC Psychiatry. (2024) 24:884. doi: 10.1186/s12888-024-06351-5, 39633344 PMC11616291

[16] Shan W Peng X Tan W Zhou Z Xie H Wang S. Prevalence of insomnia and associations with depression, anxiety among adults in Guangdong, China: a large-scale cross-sectional study. Sleep Med. (2024) 115:39–47. doi: 10.1016/j.sleep.2024.01.023, 38330694

[17] Maggi G Vitale C Cerciello F Santangelo G. Sleep and wakefulness disturbances in Parkinson’s disease: a meta-analysis on prevalence and clinical aspects of REM sleep behavior disorder, excessive daytime sleepiness and insomnia. Sleep Med Rev. (2023) 68:101759. doi: 10.1016/j.smrv.2023.101759, 36708642

[18] Gammoh O Ennab W. The prevalence and correlates of PTSD, insomnia, and fatigue among people with epilepsy during Oct.7th war on Gaza: a study from Jordan. Epilepsy Behav. (2024) 155:109768. doi: 10.1016/j.yebeh.2024.109768, 38636138

[19] Hawlader MDH Dalal K Sabrina F Faruq MFI Munaf NB Hossain A . Prevalence and factors associated with insomnia among firefighting personnel in Dhaka division, Bangladesh. BMC Public Health. (2025) 25:2665. doi: 10.1186/s12889-025-23919-2, 40770701 PMC12326870

[20] Rahman M Khan M Fattah T Tasnim Z Das PR Prattay KMR . Prevalence and associated risk factors for depression, anxiety, insomnia, and loneliness among the geriatric population in Bangladesh: a cross-sectional study. BMC Psychiatry. (2025) 25:1102. doi: 10.1186/s12888-025-07500-0, 41254547 PMC12625414

[21] Liu C Pan W Li L Li B Ren Y Ma X. Prevalence of depression, anxiety, and insomnia symptoms among patients with COVID-19: a meta-analysis of quality effects model. J Psychosom Res. (2021) 147:110516. doi: 10.1016/j.jpsychores.2021.110516, 34023580 PMC8129994

[22] Kadl A Davis EM Oliver SF Lazoff SA Popovich J Atya AAE . Prevalence and associations of insomnia after COVID-19 infection. J Clin Sleep Med. (2025) 21:383–91. doi: 10.5664/jcsm.11420, 39436395 PMC11789262

[23] Sun H-L Chen P Bai W Zhang L Feng Y Su Z . Prevalence and network structure of depression, insomnia and suicidality among mental health professionals who recovered from COVID-19: a national survey in China. Transl Psychiatry. (2024) 14:227. doi: 10.1038/s41398-024-02918-8, 38816419 PMC11139988

[24] Xu Y-M Li C Zhu R Zhong B-L. Prevalence and correlates of insomnia symptoms in older Chinese adults during the COVID-19 outbreak: a classification tree analysis. J Geriatr Psychiatry Neurol. (2022) 35:223–8. doi: 10.1177/08919887221078561, 35245996 PMC8899830

[25] Lelisho ME Wotale TW Tareke SA. Prevalence and associated factors of insomnia symptoms during the COVID-19 pandemic lockdown among Mettu town residents. PLoS One. (2023) 18:e0279624. doi: 10.1371/journal.pone.0279624, 36917577 PMC10013898

[26] Meadows RJ Paskett ED Bower JK Kaye GL Lemeshow S Harris RE. Socio-demographic differences in the dietary inflammatory index from National Health and nutrition examination survey 2005–2018: a comparison of multiple imputation versus complete case analysis. Public Health Nutr. (2024) 27:e184. doi: 10.1017/S1368980024001800, 39327915 PMC11504956

[27] Bastien CH Vallières A Morin CM. Validation of the insomnia severity index as an outcome measure for insomnia research. Sleep Med. (2001) 2:297–307. doi: 10.1016/s1389-9457(00)00065-4 11438246

[28] Chung K-F Kan KK-K Yeung W-F. Assessing insomnia in adolescents: comparison of insomnia severity index, Athens insomnia scale and sleep quality index. Sleep Med. (2011) 12:463–70. doi: 10.1016/j.sleep.2010.09.019, 21493134

[29] Wong ML Lau KNT Espie CA Luik AI Kyle SD Lau EYY. Psychometric properties of the sleep condition Indicator and insomnia severity index in the evaluation of insomnia disorder. Sleep Med. (2017) 33:76–81. doi: 10.1016/j.sleep.2016.05.019, 28449911

[30] Yu DSF. Insomnia severity index: psychometric properties with Chinese community-dwelling older people. J Adv Nurs. (2010) 66:2350–9. doi: 10.1111/j.1365-2648.2010.05394.x, 20722803

[31] Noble WS. How does multiple testing correction work? Nat Biotechnol. (2009) 27:1135–7. doi: 10.1038/nbt1209-1135, 20010596 PMC2907892

[32] Pengpid S Peltzer K. Prevalence and associated factors of insomnia symptoms among older adults in the Philippines. Psychogeriatrics. (2025) 25:e70035. doi: 10.1111/psyg.70035, 40195002

[33] Markwald RR Carey FR Kolaja CA Jacobson IG Cooper AD Chinoy ED. Prevalence and predictors of insomnia and sleep medication use in a large tri-service US military sample. Sleep Health. (2021) 7:675–82. doi: 10.1016/j.sleh.2021.08.002, 34690109

[34] Hu N Wang C Liao Y Dai Q Cao S. Smoking and incidence of insomnia: a systematic review and meta-analysis of cohort studies. Public Health. (2021) 198:324–31. doi: 10.1016/j.puhe.2021.07.012, 34507139

[35] Huang J Shi P Zhao Y Zhang H Gao T Wang X. Associations between smoking, sex steroid hormones, trouble sleeping, and depression among U.S. adults: a cross-sectional study from NHANES (2013–2016). BMC Public Health. (2024) 24:1541. doi: 10.1186/s12889-024-19045-0, 38849814 PMC11157951

[36] Page S Gibson M Munafò MR Khouja J Richmond RC. Exploring the role of nicotine and smoking in sleep behaviours: a multivariable Mendelian randomisation study. Epidemiology. (2024). doi: 10.1101/2024.08.01.24311349

