# Supplementary material for: Prevalence and associated factors of insomnia among adults in Anhui Province, China
Source: Front Public Health. 2026 May 8;14:1794419. doi: 10.3389/fpubh.2026.1794419 (PMC13193923; doi:10.3389/fpubh.2026.1794419)
Supplement: Supplementary file 1 [file Data_Sheet_1.docx]

| Supplementary Table S1 Prevalence and Odds Ratios for Insomnia by ISI Cut-off Values | | | | | | | | | | | | |
| --- | --- | --- | --- | --- | --- | --- | --- | --- | --- | --- | --- | --- |
| Variable | Num. | Cut-off value = 8 | | |  | Cut-off value =10 | | |  | Cut-off value = 15 | | |
|  |  | Insomnia Rate(%, n) | *OR*(95%*CI*) | *P* |  | Insomnia Rate(%, n) | *OR*(95%*CI*) | *P* |  | Insomnia Rate(%, n) | *OR*(95%*CI*) | *P* |
| Total | 8317 | 10.64(885) |  |  |  |  |  |  |  |  |  |  |
| Regional Distribution |  |  |  | <0.001 |  |  |  | 0.003 |  |  |  | 0.004 |
| Northern Anhui | 3679 | 9.05(333) | 1 |  |  | 5.55(204) | 1 |  |  | 1.63(60) | 1 |  |
| Central Anhui | 2797 | 11.55(323) | 1.312(1.116-1.542) | <0.001 |  | 7.11(199) | 1.305(1.066-1.597) | 0.010 |  | 2.79(78) | 1.730(1.231-2.432) | 0.002 |
| Southern Anhui | 1841 | 12.44(229) | 1.427(1.194-1.706) | <0.001 |  | 7.77(143) | 1.435(1.150-1.790) | 0.001 |  | 2.66(49) | 1.649(1.126-2.416) | 0.010 |
| Gender |  |  |  | <0.001 |  |  |  | <0.001 |  |  |  | 0.003 |
| Male | 3955 | 8.90(352) | 1 |  |  | 5.26(208) | 1 |  |  | 1.74(69) | 1 |  |
| Female | 4362 | 12.22(533) | 1.425(1.236-1.642) | <0.001 |  | 7.75(338) | 1.513(1.266-1.809) | <0.001 |  | 2.71(118) | 1.566(1.160-2.114) | 0.003 |
| Age |  |  |  |  |  |  |  |  |  |  |  |  |
| 18–<40 | 2650 | 5.58(148) | 1 |  |  | 3.02(80) | 1 |  |  | 1.06(28) | 1 |  |
| 40–<60 | 3315 | 10.92(362) | 2.072(1.699-2.527) | <0.001 |  | 6.49(215) | 2.228(1.715-2.895) | <0.001 |  | 1.99(66) | 1.902(1.219-2.969) | 0.005 |
| ≥60 | 2352 | 15.94(375) | 3.207(2.627-3.913) | <0.001 |  | 10.67(251) | 3.838(2.965-4.968) | <0.001 |  | 3.95(93) | 3.855(2.517-5.904) | <0.001 |
| Education Level |  |  |  |  |  |  |  |  |  |  |  |  |
| Junior High School or Below | 6429 | 11.70(752) | 1 | <0.001 |  | 7.42(477) | 1 | <0.001 |  | 2.60(167) | 1 | <0.001 |
| High School/Vocational High School/Technical Secondary School | 1011 | 6.13(62) | 0.493(0.377-0.645) | <0.001 |  | 3.12(32) | 0.408(0.283-0.587) | <0.001 |  | 0.89(9) | 0.337(0.172-0.661) | 0.002 |
| College/Undergraduate or Above | 876 | 8.11(71) | 0.666(0.516-0.859) | 0.002 |  | 4.22(37) | 0.550(0.391-0.775) | <0.001 |  | 1.26(11) | 0.477(0.258-0.881) | 0.018 |
| Occupation |  |  |  | <0.001 |  |  |  | 0.009 |  |  |  | 0.114 |
| Civil Servant/Professional Technician | 766 | 5.35(41) | 1 |  |  | 3.39(26) | 1 |  |  | 0.78(6) | 1 |  |
| Business/Service Industry | 1172 | 9.73(114) | 1.905(1.317-2.756) | <0.001 |  | 6.06(71) | 1.835(1.160-2.904) | 0.009 |  | 2.47(29) | 3.214(1.328-7.778) | 0.010 |
| Farmers/Workers | 6016 | 11.85(713) | 2.378(1.719-3.288) | <0.001 |  | 7.30(439) | 2.240(1.497-3.352) | <0.001 |  | 2.48(149) | 3.217(1.417-7.301) | 0.005 |
| Other | 360 | 4.72(17) | 0.876(0.491-1.565) | 0.656 |  | 2.78(10) | 0.813(0.388-1.705) | 0.584 |  | 0.83(3) | 1.064(0.265-4.280) | 0.930 |
| Marital Status |  |  |  | <0.001 |  |  |  | <0.001 |  |  |  | 0.002 |
| Unmarried | 860 | 6.16(53) | 1 |  |  | 3.37(29) | 1 |  |  | 1.40(12) | 1 |  |
| Married | 6925 | 10.54(730) | 1.794(1.345-2.394) | <0.001 |  | 6.47(448) | 1.982(1.352-2.905) | <0.001 |  | 2.19(152) | 1.586(0.877-2.867) | 0.127 |
| Divorced/Widowed/Other | 532 | 19.17(102) | 3.612(2.540-5.135) | <0.001 |  | 12.97(69) | 4.270(2.727-6.687) | <0.001 |  | 4.32(23) | 3.193(1.575-6.473) | 0.001 |
| Chronic Illness |  |  |  | <0.001 |  |  |  | <0.001 |  |  |  | <0.001 |
| No | 4845 | 5.84(283) | 1 |  |  | 3.05(148) | 1 |  |  | 0.89(43) | 1 |  |
| Yes | 3472 | 17.34(602) | 3.381(2.914-3.924) | <0.001 |  | 11.46(398) | 4.109(3.384-4.989) | <0.001 |  | 4.15(144) | 4.832(3.427-6.812) | <0.001 |
| Personal Monthly Income (CNY) |  |  |  | <0.001 |  |  |  | <0.001 |  |  |  | <0.001 |
| <1500 | 4227 | 14.34(606) | 1 |  |  | 9.27(392) | 1 |  |  | 3.34(141) | 1 |  |
| 1500~<3000 | 1903 | 7.83(149) | 0.508(0.421-0.613) | <0.001 |  | 4.41(84) | 0.452(0.355-0.576) | <0.001 |  | 1.58(30) | 0.464(0.312-0.691) | <0.001 |
| 3000~<5000 | 1533 | 4.96(76) | 0.312(0.244-0.399) | <0.001 |  | 2.74(42) | 0.276(0.199-0.381) | <0.001 |  | 0.65(10) | 0.190(0.100-0.362) | <0.001 |
| ≥5000 | 654 | 8.26(54) | 0.538(0.402-0.720) | <0.001 |  | 4.28(28) | 0.438(0.296-0.648) | <0.001 |  | 0.92(6) | 0.268(0.118-0.610) | 0.002 |
| Monthly Household Income (CNY) |  |  |  | <0.001 |  |  |  | <0.001 |  |  |  | <0.001 |
| <3000 | 2575 | 15.03(387) | 1 |  |  | 9.98(257) | 1 |  |  | 3.73(96) | 1 |  |
| 3000~<6000 | 2539 | 8.74(222) | 0.542(0.455-0.645) | <0.001 |  | 5.40(137) | 0.514(0.415-0.638) | <0.001 |  | 1.85(47) | 0.487(0.342-0.693) | <0.001 |
| 6000~<10000 | 1937 | 8.00(155) | 0.492(0.404-0.599) | <0.001 |  | 4.59(89) | 0.434(0.339-0.557) | <0.001 |  | 1.14(22) | 0.297(0.186-0.473) | <0.001 |
| ≥10000 | 1266 | 9.56(121) | 0.597(0.481-0.742) | <0.001 |  | 4.98(63) | 0.472(0.355-0.628) | <0.001 |  | 1.74(22) | 0.457(0.286-0.729) | 0.001 |
| Smoker |  |  |  | 0.012 |  |  |  | 0.006 |  |  |  | 0.073 |
| No | 6441 | 11.10(715) | 1 |  |  | 6.97(449) | 1 |  |  | 2.41(155) | 1 |  |
| Yes | 1876 | 9.06(170) | 0.798(0.669-0.951) | 0.012 |  | 5.17(97) | 0.728(0.581-0.912) | 0.006 |  | 1.71(32) | 0.704(0.479-1.033) | 0.073 |
| Alcohol Consumer |  |  |  | 0.939 |  |  |  | 0.194 |  |  |  | 0.907 |
| No | 6062 | 10.66(646) | 1 |  |  | 6.78(411) | 1 |  |  | 2.26(137) | 1 |  |
| Yes | 2255 | 10.60(239) | 0.994(0.850-1.163) | 0.939 |  | 5.99(135) | 0.876(0.716-1.070) | 0.194 |  | 2.22(50) | 0.981(0.707-1.361) | 0.907 |
| Self-Rated Health Status |  |  |  | <0.001 |  |  |  | <0.001 |  |  |  | <0.001 |
| Good | 5474 | 4.91(269) | 1 |  |  | 2.76(151) | 1 |  |  | 0.66(36) | 1 |  |
| Fair | 2307 | 17.60(406) | 4.132(3.512-4.863) | <0.001 |  | 9.97(230) | 3.904(3.160-4.823) | <0.001 |  | 3.08(71) | 4.796(3.202-7.184) | <0.001 |
| Poor | 536 | 39.18(210) | 12.464(10.080-15.413) | <0.001 |  | 30.78(165) | 15.678(12.277-20.021) | <0.001 |  | 14.93(80) | 26.501(17.679-39.725) | <0.001 |

| Supplementary Table 2 Multivariate Association Analysis of Insomnia Based on ISI Cut-off Thresholds | | | | | | | | | | | | |
| --- | --- | --- | --- | --- | --- | --- | --- | --- | --- | --- | --- | --- |
| Variable | Num. | Cut-off value = 8 (*R^2^* = 0.173) | | |  | Cut-off value =10 (*R^2^* = 0.173) | | |  | Cut-off value = 15 (*R^2^* = 0.180) | | |
|  |  | Insomnia Rate(%, n) | *OR*(95%*CI*) | *P* |  | Insomnia Rate(%, n) | *OR*(95%*CI*) | *P* |  | Insomnia Rate(%, n) | *OR*(95%*CI*) | *P* |
| Regional Distribution |  |  |  | <0.001 |  |  |  | <0.001 |  |  |  | 0.002 |
| Northern Anhui | 3679 | 9.05(333) | 1 |  |  | 5.55(204) | 1 |  |  | 1.63(60) | 1 |  |
| Central Anhui | 2797 | 11.55(323) | 1.450(1.218-1.726) | <0.001 |  | 7.11(199) | 1.368(1.106-1.692) | 0.004 |  | 2.79(78) | 1.793(1.262-2.547) | 0.001 |
| Southern Anhui | 1841 | 12.44(229) | 1.612(1.329-1.955) | <0.001 |  | 7.77(143) | 1.514(1.200-1.912) | <0.001 |  | 2.66(49) | 1.732(1.169-2.566) | 0.006 |
| Gender |  |  |  | <0.001 |  |  |  | <0.001 |  |  |  |  |
| Male | 3955 | 8.90(352) | 1 |  |  | 5.26(208) | 1 |  |  | 1.74(69) |  |  |
| Female | 4362 | 12.22(533) | 1.365(1.170-1.593) | <0.001 |  | 7.75(338) | 1.498(1.243-1.805) | <0.001 |  | 2.71(118) |  |  |
| Chronic Illness |  |  |  | <0.001 |  |  |  | <0.001 |  |  |  | <0.001 |
| No | 4845 | 5.84(283) | 1 |  |  | 3.05(148) | 1 |  |  | 0.89(43) | 1 |  |
| Yes | 3472 | 17.34(602) | 1.745(1.469-2.072) | <0.001 |  | 11.46(398) | 2.133(1.715-2.653) | <0.001 |  | 4.15(144) | 1.970(1.339-2.900) | <0.001 |
| Personal Monthly Income (CNY) |  |  |  | <0.001 |  |  |  |  |  |  |  |  |
| <1500 | 4227 | 14.34(606) | 1 |  |  | 9.27(392) | - | - |  | - | - | - |
| 1500~<3000 | 1903 | 7.83(149) | 0.798(0.651-0.978) | 0.029 |  | 4.41(84) | - | - |  | - | - | - |
| 3000~<5000 | 1533 | 4.96(76) | 0.599(0.459-0.783) | <0.001 |  | 2.74(42) | - | - |  | - | - | - |
| ≥5000 | 654 | 8.26(54) | 1.034(0.750-1.424) | 0.840 |  | 4.28(28) | - | - |  | - | - | - |
| Self-Rated Health Status |  |  |  | <0.001 |  |  |  | <0.001 |  |  |  | <0.001 |
| Good | 5474 | 4.91(269) | 1 |  |  | 2.76(151) | 1 |  |  | 0.66(36) | 1 |  |
| Fair | 2307 | 17.60(406) | 3.233(2.715-3.851) | <0.001 |  | 9.97(230) | 3.003(2.399-3.760) | <0.001 |  | 3.08(71) | 3.791(2.476-5.805) | <0.001 |
| Poor | 536 | 39.18(210) | 8.152(6.438-10.323) | <0.001 |  | 30.78(165) | 10.373(7.932-13.567) | <0.001 |  | 14.93(80) | 18.273(11.656-28.648) | <0.001 |
